# Receptor Usage and the Pathogenesis in Acute and Chronic Virus Infections

**DOI:** 10.3389/fmicb.2012.00289

**Published:** 2012-08-08

**Authors:** Yasuko Tsunetsugu-Yokota, Kazutaka Terahara

**Affiliations:** ^1^Department of Immunology, National Institute of Infectious DiseasesTokyo, Japan

In the first phase of the viral life cycle, the virus enters cells using a specific cell surface receptor. Many viruses use multiple receptors: some of which are unique to a certain cell type, whereas others are found in many cell types. After the virus enters into cells, various cellular proteins may interact with it; some support virus replication, while others inhibit it. Once virus succeeds to establish its life cycle in the target cell, the progeny viruses disseminate within the tissues or systemically via viremia. The intrinsic pro- or anti-viral cellular machinery differs among cell types. Thus, depending on the receptors used, the viral cell tropism is determined, resulting in the characteristic distribution of virus-infected cells/tissues and the disease outcome *in vivo*.

How viral cell tropism, determined by the receptor used, can affect the disease outcome in acute and chronic virus infection is a major subject under extensive investigation in Virology. To understand the mechanism which causes human diseases by viruses, that is, viral pathogenesis, we have studied various aspects of virus infection at a cell/tissue level or using animal models. In this context, the recent development of reverse genetics allows us to visualize virus-infected cells/tissues or even the virus itself. By applying such manipulated viruses to animal models, it is also possible to analyze the dynamics of virus infection *in vivo*.

In this Research Topic, we selected on studies connecting virus receptor usage and the pathogenesis of various viruses causing acute or chronic infection. Eventually, it could expand to cover the receptor-pathogenesis relationship in various acute and chronic virus infection. This research topic comprises an original research article on HIV-1, two opinions articles on the hepatitis C virus (HCV) and norovirus, while the remaining review articles on HTVL-1, measles virus (MV), mouse hepatitis virus (MHV), influenza virus, HCV, and enterovirus (EV) provide overviews on various aspects of viral pathogenesis. In all these review/opinion articles, at least one comprehensive table or figure is incorporated so that readers who are unfamiliar with these viruses can get a message at a glance.

With regards to the cell tropism of HIV-1, Terahara et al. (2012) presented his recent study using CCR5-tropic and CXCR4-tropic HIV-1 with distinct fluorescent reporter. These HIV-1s allowed us to detect HIV-infected cells at a different stage of infection and to evaluate the level of virus replication in CD4^+^ T cells with distinct differentiation phenotype including CCR5^+^ memory. In contrast, a receptor for HTLV-1 and related pathogenesis is still intriguing issue, which is described by Hoshino (2012) in his extensive review.

The two reviews on the MV (Kato et al., 2012; Takeda et al., 2012) were published at a very appropriate time as a third receptor for MV entry into epithelial cells, nectin 4, had just been discovered (Muhlebach et al., 2011; Noyce et al., 2011). Here, Kato et al. (2012) focused on the receptor usage of MV *in vivo* which may influences the disease outcome using monkey models, while Takeda et al. (2012) discussed about the dual-tropic nature of MV using SLAM and nectin 4 expressed in immune cells and epithelial cells, respectively.

Ito et al. (2012) addressed the importance of B cells as a reservoir for persistent HCV infection. In two reviews on HCV, Moriishi and Matsuura (2012) overviewed a current research focus on lipid components for the HCV pathogenesis, while Shoji et al. (2012) discussed about glucose metabolic disorders associated with HCV infection.

Nishimura and Shimizu (2012) and Yamayoshi et al. (2012), both of whom successfully identified two novel receptors for EV, overviewed the current knowledge about receptor usage and various diseases associated with EV infection. For the coronavirus, Taguchi and Hirai-Yuki (2012) overviewed studies on the receptor and related cellular factors for MHV, which may contribute to the mouse susceptibility to MHV infection.

As regards to the virus recognizing sugar moieties, Ramos and Fernandez-Sesma (2012) provided insights about the interaction of influenza A virus with sialic acid receptors on immune cells with special reference to the innate immune response. Shirato (2012) described about the norovirus with distinct genotypes which recognize a specific structure of sugar chain.

We will learn by these articles the fact that to identify a receptor is the first important step to know a virus, but many questions remain in order to fully understand human diseases caused by viruses. I would like to express my cordial thanks to all the contributors for this topic. I hope readers find the content interesting, but most importantly, that the information will prove very useful for future research.
